# BCheM: a framework integrating molecular attributes and behavioral representations for precise prediction of drug resistance across multiple disease contexts

**DOI:** 10.3389/fbinf.2026.1838904

**Published:** 2026-05-19

**Authors:** Wenxuan Yu, Zhenyu Wang, Beichen Shen, Ziqi Xia, Xuemei Hu

**Affiliations:** 1 School of Data Science, Master of Science in Data Science, Lingnan University, Hongkong, China; 2 School of Automation and Electrical Engineering, Lanzhou University of Technology, Lanzhou, China; 3 School of Animal Science and Technology, Qingdao Agricultural University, Qingdao, China; 4 School of Integrative Medicine, Nanjing University of Chinese Medicine, Nanjing, China; 5 College of Information Engineering, Northwest A&F University, Xianyang City, China

**Keywords:** drug resistance, drug sensitivity, graph convolutional neural networks, graph representation learning, personalized treatment

## Abstract

**Introduction:**

In clinical medicine, the response of diseases to drugs directly affects treatment efficacy and patient prognosis. Accurate prediction of drug responses can facilitate personalized treatment planning, enable the selection of effective medications, and reduce adverse effects. However, existing computational methods are often limited to single disease types or rely solely on molecularlevel information, making it difficult to simultaneously capture the semantic attribute features of diseases and drugs, as well as their behavioral patterns within association networks. Therefore, effectively integrating multimodal attribute information with complex relational structures for drug resistance prediction across multiple disease contexts remains a challenging problem.

**Methods:**

To address this issue, we propose a novel disease-drug resistance prediction framework, BCheM. First, a disease-drug resistance network is constructed based on experimentally validated drug response data, and rich attribute features are obtained by incorporating drug SMILES representations and disease semantic descriptions. Then, pre-trained models are employed to encode disease semantics and drug molecular structures, enabling deep representation of molecular attributes. Furthermore, a graph representation learning model that integrates Chebyshev graph convolution with a multi-head attention mechanism is developed to model and fuse behavioral features within the disease-drug network. Finally, a classifier is applied to predict potential disease-drug resistance associations.

**Results:**

Experimental results and case studies demonstrate that BCheM achieves superior performance across multiple evaluation metrics, exhibiting strong predictive capability and practical applicability.

**Discussion:**

This study provides an effective graph-based modeling approach for drug response prediction in multi-disease contexts.

## Highlights

- We constructed a comprehensive multi-type disease-drug resistance dataset that includes molecular attributes.
- We developed a multi-type disease-drug resistance network (DDRN), enabling the extraction of deep insights into the impacts of diseases and drugs through graph-level modeling.
- We proposed the BCheM framework, which enables efficient prediction of multi-type disease-drug resistance.

## 1 Introduction

The study of disease response to drugs, particularly in terms of drug resistance, holds significant importance in modern medicine, as it directly affects individual treatment outcomes. Drug resistance refers to the decreased sensitivity of pathogens, tumor cells, or the human body to a drug, rendering standard doses ineffective in producing the desired therapeutic effect (Davies and Davies, 2010). Drug sensitivity, on the other hand, refers to the heightened responsiveness of pathogens, cells, or the body to a drug, meaning that when exposed to a certain drug, the pathogen or cells are rapidly and effectively inhibited or destroyed (Garnett et al., 2012; Costello et al., 2014). For drug-sensitive pathogens or cells, lower doses of medication typically yield favorable therapeutic outcomes without the need for increased dosage or prolonged treatment duration, whereas resistance requires the opposite approach. Understanding the mechanisms behind drug resistance in disease not only aids in improving the success rate of clinical treatments but also contributes to the development of more effective drugs and the reduction of drug resistance.

Variations in drug response stem from a complex array of biological mechanisms, including individual genetic differences, genetic mutations in pathogens or tumor cells, environmental factors, and differences in drug metabolism. Since the introduction of antibiotics in the early 20th century, the issue of drug resistance has gradually emerged, particularly in the treatment of infectious diseases and cancer (Nanayakkara et al., 2021). With the widespread use of antibiotics, many bacteria have developed resistance to multiple antibiotics through mechanisms such as genetic mutations and horizontal gene transfer. The spread of drug-resistant pathogens not only complicates treatment but also forces physicians to resort to more toxic and expensive alternative drugs, thereby increasing healthcare costs and treatment risks. In treatment, drug sensitivity is a crucial factor for developing personalized therapeutic strategies. For example, in cancer therapy, the effectiveness of targeted drugs often depends on specific genetic mutations in tumor cells, and only those tumor cells carrying the mutation are sensitive to the drug (Westover et al., 2018). Therefore, investigating disease responses to drugs can optimize therapeutic regimens and improve patient survival rates.

However, the high development costs and long timelines are significant obstacles to drug development. The process of bringing a new drug from research to market typically requires an investment of billions of dollars and more than 10 years (Wouters et al., 2020). This high cost and extended timeline increase the risk of return on investment, particularly in the context of rapidly evolving drug resistance, causing many companies to hesitate in investing in new drugs (Pammolli et al., 2011). Additionally, ethical constraints pose another major challenge. The design of clinical trials must balance scientific advancement with patient rights, particularly when new drugs are involved, and it is crucial to consider the potential risks and uncertainties (Emanuel et al., 2000). The recruitment and protection of high-risk groups, along with the evaluation of long-term drug effects, are often hindered by ethical restrictions (Pace and Emanuel, 2005). On the experimental level, *in vitro* and animal models cannot fully replicate the complex physiological environment of the human body, leading to uncertainties when translating research findings into clinical applications (Kim et al., 2020). Furthermore, the genetic heterogeneity and adaptive changes of pathogens or cancer cells add to the complexity of studying drug response mechanisms (Holohan et al., 2013).

With the increase of computational power and accumulation of biological data, computational methods have been widely used in biomedical entity association prediction tasks, such as circRNA-disease association prediction (Wang Z. et al., 2026; Wei et al., 2025; Li et al., 2025), ncRNA-disease association prediction (Wang et al., 2025; Wang X. et al., 2026; Wang et al., 2024), and circRNA-miRNA interaction prediction (Wei et al., 2026a; Li et al., 2024; Wang X.-F. et al., 2023; Wang et al., 2022; Wei et al., 2026b). Meanwhile, computational methods have broad application prospects in drug development. These approaches, by integrating bioinformatics, chemoinformatics, and artificial intelligence technologies, assist researchers in screening potential drug candidates and predicting their interactions with targets in the early stages of development (Güvenç Paltun et al., 2021; Chen and Zhang, 2021; Li et al., 2022).

For example, in cancer drug response prediction, Wang et al. proposed GADRP, which combines graph convolutional neural networks and autoencoders to predict potential effective anticancer drugs (Wang H. et al., 2023); Liu et al. introduced GraphCDR, leveraging graph contrastive learning to predict the response of cancer cell lines to therapeutic drugs (Liu et al., 2022). In the area of miRNA-related drug resistance, Zheng et al. proposed NASMDR, which integrates efficient neural architecture search and graph isomorphism networks to predict drug resistance-associated miRNAs (Zheng et al., 2022); Wei et al. developed GCFMCL, combining graph collaborative filtering and multi-view contrastive learning to predict miRNA drug sensitivity (Wei et al., 2023). Moreover, computational methods have also been applied to drug-drug interaction (DDI) modeling (Zhao et al., 2024) to predict synergistic effects between different drugs. For instance, Li et al. proposed SNRMPACDC, utilizing a Siamese network and random matrix projection to predict anticancer synergistic drug combinations (Li et al., 2023); Han et al. developed MCFF-MTDDI, constructing a multi-channel feature fusion framework to predict various types of drug interactions (Han et al., 2023); Su et al. introduced the TIGER framework, combining relation-aware heterogeneous graph transformers to predict DDI (Su et al., 2024); Ren et al. proposed BioDKG-DDI, using a drug knowledge graph based on integrated biochemical information to predict potential drug interactions (Ren et al., 2022). These studies, by integrating experimental data with computational predictions, assist researchers in making more informed decisions at various stages of drug development, thereby improving efficiency and reducing failure rates.

Although existing studies have made considerable progress in predicting disease-related drug resistance, several critical limitations remain. First, due to constraints in data availability and research scope, most current methods focus on specific disease types or single molecular levels, such as cancer or fungal infections with high resistance, lacking a unified modeling framework for multi-disease scenarios. Second, the majority of approaches primarily rely on molecular structural information or local features, making it difficult to simultaneously capture the attribute features of diseases and drugs, as well as their behavioral characteristics within interaction networks. This insufficient capability to model multimodal information and complex relational structures limits the generalization ability and predictive performance of existing methods in multi-disease contexts.

To address these challenges, we propose BCheM, a novel disease–drug resistance prediction framework designed for multi-disease scenarios. First, a disease–drug resistance network (DDRN) is constructed based on experimentally validated drug response data. To enrich node attributes and enable multimodal representation, drug SMILES representations and disease semantic descriptions are incorporated to capture comprehensive attribute features. Then, pre-trained language models are employed to encode disease semantics and drug molecular structures, generating high-quality representations of molecular attributes. Furthermore, a graph representation learning model integrating Chebyshev graph convolution and a multi-head attention mechanism is developed to model behavioral features and propagate information within the disease-drug network, thereby achieving deep fusion of attribute and structural information. Finally, a classifier is applied to predict potential disease-drug resistance associations. Experimental results demonstrate that BCheM outperforms existing methods across multiple evaluation metrics, exhibiting strong predictive performance and robustness. In addition, case studies on Fluorouracil and Doxorubicin show that the predicted resistance relationships are supported by existing literature, further validating the effectiveness and practical applicability of the proposed framework.

## 2 Materials and methods

### 2.1 Materials

To address the issue of limited data on drug resistance for multiple diseases in existing research, we collected data from publicly available databases and utilized large language models to construct a comprehensive disease-drug resistance dataset with extensive attribute information. Specifically, we downloaded drug response data for 395 disease types from the DRESIS database (Sun et al., 2023). Proposed by Sun et al., the DRESIS database is the first comprehensive database of drug resistance, encompassing the widest range of disease types documented in existing databases, all supported by clinical and experimental validation data.

Subsequently, we performed a rigorous screening and deduplication of the downloaded data, resulting in 3,261 reaction data between 395 diseases and 1,391 drugs. This dataset includes 2,041 instances of drug resistance and 1,220 instances of drug sensitivity, along with the SMILES representations for the 1,391 drugs. We retained the associations of drug resistance between the 395 diseases and 1,391 drugs, using non-redundant instances of drug sensitivity as negative samples. To enhance the attribute representation of the 395 diseases, we generated high-quality textual semantic descriptions using GPT-4.0. The generation process consists of the following steps:

1. Input Disease Name: Each disease name was provided as a prompt to the model, instructing GPT-4.0 to generate a detailed description of the disease. The prompt included instructions such as, “Please generate a comprehensive semantic description of the disease, including its symptoms, etiology, and impact.”
2. Generating Semantic Descriptions: Leveraging the medical knowledge acquired during its pre-training, GPT-4.0 produced semantic description texts based on the provided disease names. Each description encompassed symptoms, causes, and clinical characteristics, ensuring the completeness and medical relevance of the information.
3. Data Quality Control: To ensure that the generated descriptions met scientific standards and accuracy, the outputs underwent a review and filtering process to eliminate any irrelevant content or inaccuracies.

The generated disease descriptions are structured texts, with each description consisting of several hundred words to provide a thorough overview of the disease. The format of the dataset is as follows (Equation 1):

$$Disease\text{ attributes}={\left\{\left(Disease_{i},Description_{i}\right)\right\}}_{i}^{395}$$ (1)

These descriptive texts were subsequently utilized for the extraction of node attribute features.

### 2.2 Method

**FIGURE 1:**
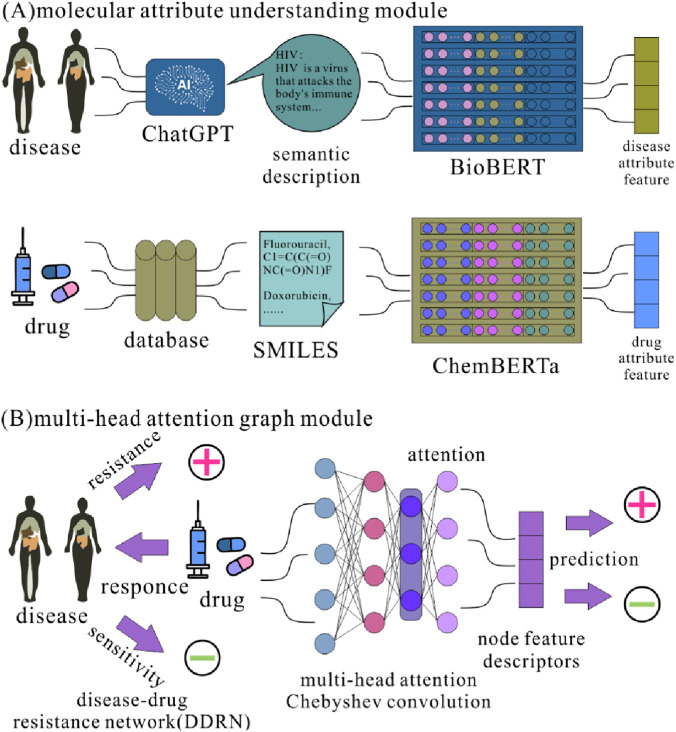
The architecture of BCheM.

Figure 1 illustrates the architecture of BCheM. BCheM consists of two modules: molecular attribute understanding and multi-head attention graph, which comprehensively capture insights into molecular attributes and behavioral characteristics from the perspectives of disease semantics, drug molecular representation, and DDRN. Specifically, BCheM first employs pre-trained BioBERT (Lee et al., 2020) and ChemBERTa (Chithrananda et al., 2020) for disease semantics and drug SMILES representation, respectively, to achieve a comprehensive understanding of molecular attributes. Then the molecular attribute descriptors serve as initial features, combined with disease-drug resistance to construct an information-rich disease-drug resistance graph model. Nest utilizing Chebyshev graph convolution with a multi-head attention mechanism, BCheM learns in-depth insights into molecular behaviors within the disease-drug resistance graph. Ultimately, high-representative descriptors that encapsulate molecular attributes and behavioral characteristics are used as unique identifiers for the molecules, which are then input into an advanced classifier for training to predict unknown possible drug resistance of the disease.

## 3 Molecular attribute understanding

In this experiment, we constructed a molecular attribute understanding module to effectively capture the hidden features within molecular attributes. This module learns feature insights from both disease semantics and drug molecular structures, providing more accurate and in-depth input features for downstream drug response prediction tasks. Specifically, we employed the pre-trained BioBERT (Lee et al., 2020) and ChemBERTa (Chithrananda et al., 2020) models to generate the attribute features of the nodes. The selection of these models is based on their strengths in biomedical text understanding and chemical molecular representation, ensuring comprehensive modeling of diseases and drugs from both semantic and structural perspectives.

For the semantic descriptions of diseases, we utilized the BioBERT model (Bidirectional Encoder Representations from Transformers for Biomedical Text Mining), which is initialized with the weights of the BERT model and pre-trained on biomedical corpora, including PubMed abstracts and full-text articles from PMC. BioBERT has demonstrated exceptional performance in popular biomedical text mining tasks such as Named Entity Recognition (NER), Relation Extraction (RE), and Question Answering (QA). This pre-training enables BioBERT to effectively extract semantic information from disease descriptions, thereby enhancing the modeling of disease nodes within our framework.

We represent the textual descriptions of diseases as an input sequence, denoted as Equation 2:

$$D_{disease}=\left\{d_{1},d_{2},\ldots ,d_{n}\right\}$$ (2)

Where *d*_*i*_ represents the *ith* word in the semantic description of the disease, and *n* denotes the length of the input text. Next, we employ BioBERT to map the input disease semantic description *D* to word vector embeddings, as shown in Equation 3.

$$W_{D}=BioBERT\;\left(D_{disease}\right)$$ (3)

BioBERT uses a bidirectional Transformer structure to model the complex dependencies between words in the text and extract context-related semantic information from the input text data to generate rich semantic features. Among them, *W*_*D*_ is the word vector representation of each word in the text description, and these word vectors contain semantic information extracted from the context. We select the output of the [CLS] tag as the semantic feature vector of the overall disease description, expressed as shown in Equation 4:

$$R_{D}=W_{D}^{\left[CLS\right]}$$ (4)

The vector R_D_ will serve as the attribute feature insights for the disease node, to be utilized in subsequent graph-level modeling of disease-drug responses.

For the structure descriptions of the drug, we introduce the pre-trained ChemBERTa to learn the contextual relationship representations between atoms for drug SMILES (Simplified Molecular Input Line Entry System). ChemBERTa is a pre-trained model specifically designed for processing molecular representations, built upon the BERT architecture and trained using SMILES. Through pre-training on a large-scale chemical molecular dataset, ChemBERTa is capable of capturing the intricate patterns of atoms and their bonding relationships within SMILES strings, thereby generating structural features of the molecules.

We utilize the SMILES representation of drugs as the input text, formatted as follows (Equation 5):

$$T_{drug}=\left\{t_{1},t_{2},\ldots ,t_{n}\right\}$$ (5)

SMILES is a concise molecular representation in which each character corresponds to a specific atom or chemical bond. Thus, unlike the semantic descriptions of diseases, *t*_*i*_ denotes the *ith* character in the SMILES, and *n* represents the length of the input SMILES. ChemBERTa encodes the input SMILES to generate an embedded representation of the molecular structure (Equation 6):

$$W_{T}=ChemBERTa\;\left(T_{drug}\right)$$ (6)

Using ChemBERTa, we obtained embedding vectors for each character in the SMILES, representing the atomic and bonding characteristics of the drug molecule. Similarly, we selected the output vector corresponding to the [CLS] token as the feature vector for the representation of the entire drug molecule, as shown in Equation 7:

$$R_{T}=W_{T}^{\left[CLS\right]}$$ (7)

The feature vector *R*_*T*_ serves as the attribute for the drug node, facilitating further analysis within the disease-drug response graph.

## 4 Multi-head attention graph module

BCheM learns disease and drug-specific insights through its molecular attribute understanding module. To effectively leverage molecular attributes and known disease-drug response associations, we construct a multi-head attention graph module that enables graph-level modeling of this prior knowledge, capturing insights into molecular behavior within the interaction network.

Specifically, BCheM incorporates Chebyshev convolution (Defferrard et al., 2016) as the foundational graph convolution method and integrates a multi-head attention mechanism to enhance feature extraction capabilities. By inputting the initial node features and the adjacency matrix, the model enables feature propagation and enhancement of node attributes within the interaction graph, ultimately yielding more precise node feature representations.

Chebyshev convolution is a type of graph convolution based on spectral graph convolution. It approximates the graph Laplacian operator using Chebyshev polynomials, thereby avoiding the direct computation of Laplacian eigendecomposition, which is computationally expensive for large graphs. The core idea of Chebyshev convolution is to represent the convolution kernel on the graph using Chebyshev polynomials.

In general, graph convolution propagates node features based on the graph’s Laplacian matrix. For a given graph G=(V, E), the normalized symmetric Laplacian matrix is defined as Equation 8:

$$LA=I-D^{-1/2}AD^{-1/2}$$ (8)

As shown in Equation 9, where *A* is the adjacency matrix of the input disease-drug response association, and *D* is the degree matrix:

$$D_{ii}=\sum_{j}A_{ij}$$ (9)

The core idea of Chebyshev convolution is to approximate the graph convolution operation using Chebyshev polynomials. The Chebyshev polynomials *T*_*K*_ *(X)* can be expressed as follows in Equation 10:

$$T_{0}\left(x\right)=1,T_{1}\left(x\right)=x,T_{k}\left(x\right)=2xT_{k-1}\left(x\right)-T_{k-2}\left(x\right),fork\geq 2$$ (10)

Where *T*_*K*_ *(X)* is the Chebyshev polynomial of order *k*.

By utilizing Chebyshev polynomials to replace the convolution kernel, the convolution kernel in Chebyshev convolution can be represented as follows in Equation 11:

$$H^{\left(l+1\right)}=\sum_{k=0}^{k}T_{k}\left(LA\right)H^{\left(l\right)}W_{k}$$ (11)

Where *H* ^*(l)*^ is the node feature matrix of the *lth* layer.

Since simple weighted averaging or convolution may not fully capture the complex interactions between nodes, we introduce the multi-head attention mechanism (Vaswani et al., 2017) to further enhance feature extraction capabilities. This mechanism allows different node pairs to be assigned varying weights, enabling the model to capture the relationships between nodes in different contexts.

The core idea of the attention mechanism is to learn information from different “attention subspaces” through the parallel computation of multiple attention heads. Specifically, the input data undergoes multiple linear transformations to generate the query vector Q, the key vector K, and the value vector V. Then, the similarity between Q and K is calculated to derive the attention weights, which are used to perform a weighted sum on V. Finally, the outputs of multiple attention heads are concatenated to produce the final output. For an input feature vector of dimension C, the attention mechanism first projects the input features into different attention heads, where each head processes a feature dimension of dk = C/H, with H being the number of attention heads and dk representing the feature dimension processed by each attention head.

The input feature x is transformed linearly to generate query Q, key K, and value V vectors respectively (Equation 12):

$$Q=W_{Q}x,K=W_{K}x,V=W_{V}x$$ (12)

Where W_Q_, W_K_, W_V_ are the learned linear transformation matrices. The attention weight of multi-head attention is calculated as follows in Equation 13:

$$Att\;\left(Q,K,V\right)=soft\max\left(\frac{QK^{T}}{\sqrt{d_{k}}}\right)V$$ (13)

Att represents the mutual influence between nodes. The larger the value, the greater the contribution of a node to the current node. For each head, the above attention operation is calculated independently to obtain multiple different attention outputs. These outputs are then concatenated on the feature dimension:

$$MultiHead\;\left(Q,K,V\right)=concat\;\left(head_{1},head_{2},head_{3},head_{4}\right)W$$ (14)

Finally, after parallel processing by multiple attention heads, the output features will fuse information from different subspaces, improving the model’s ability to capture complex disease-drug resistance relationships.

## 5 Classification strategy

After extracting features using the Multi-Head Attention Chebyshev Convolution, we utilized node feature descriptors to predict drug-disease resistance in a binary classification task. Specifically, the prediction task for disease-drug resistance is defined as a binary classification task, where a drug is labeled as a positive sample if it exhibits resistance to disease, and as a negative sample if it is sensitive to the disease or has no association.

However, due to data collection constraints, imbalanced data is a common and significant issue in the prediction of disease-drug resistance. Typically, the ratio of positive samples to negative samples may vary significantly, leading to an imbalance in data distribution. This imbalance can cause the model to be biased towards the majority class during training, resulting in lower recognition rates for the minority class samples. To address this issue, BCheM applies resampling techniques on the training set. Specifically, within each fold of the training set, BCheM performs random oversampling on the minority class samples by randomly duplicating them to increase their proportion in the training set, thereby achieving a balanced sample effect. Finally, BCheM employed an AdaBoost classifier (Freund and Schapire, 1997) to learn from the balanced training data and predict the associations for the unseen test set.

## 6 Results

### 6.1 Evaluation criteria

To objectively and effectively evaluate the performance of BCheM in disease-drug resistance prediction, we conducted a five-fold cross-validation (5-fold CV) based on benchmark data. Specifically, in the 5-fold CV, the known disease-drug resistance data was divided into five equal subsets. In each fold, four subsets were used for feature extraction and classifier training, while one subset was used for the prediction task. This process was repeated five times until all unique subsets had been assigned prediction scores. To comprehensively assess the predictive performance, we introduced multiple evaluation criteria, including accuracy (Acc.), precision (Prec.), recall (Rec.), F1-score, area under the ROC curve (AUC), and area under the precision-recall curve (AUPR). These evaluation criteria were calculated as follows in Equations 15–18:

$$\text{Acc}.=\frac{TP+TN}{TP+TN+FP+FN}$$ (15)

$$\mathrm{Pr}ec.=\frac{TP}{TP+FP}$$ (16)

$$\text{Re}c.=\frac{TP}{TP+FN}$$ (17)

$$F1-score=\frac{2prec\times recall}{prec+recall}$$ (18)

Among them, TP and TN represent the correct predictions of drug resistance and drug sensitivity by BCheM, respectively; FP and FN represent the incorrect predictions of drug resistance and drug sensitivity by BCheM, respectively.

All experiments were conducted on a standard computing platform equipped with an Intel Core i7 processor, 32 GB RAM, and an NVIDIA GeForce RTX 4090 GPU, and implemented using the PyTorch deep learning framework.

### 6.2 Performance evaluation

**FIGURE 2:**
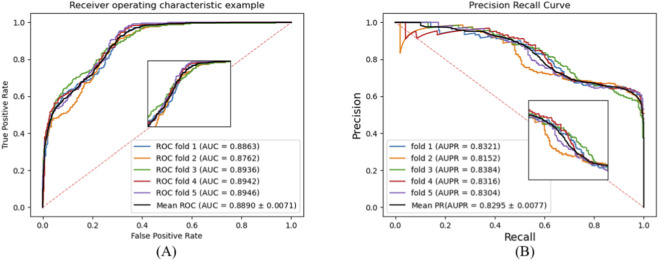
ROC and P-R curves of BCheM on the benchmark dataset. ((A) ROC curves under 5-fold CV; **(B)** P-R curves under 5-fold CV).

To validate the performance of BCheM in predicting disease-drug resistance, we conducted the 5-fold CV based on benchmark datasets, with the results recorded in Table 1. Additionally, we automatically generated Receiver operating characteristic (ROC) and Precision-recall (P-R) curves for the models in the prediction tasks, as shown in Figure 2.

**TABLE 1:** 5-fold CV prediction results of BCheM based on benchmark datasets.

BCheM	Acc	Prec.	Rec.	F1-score	AUC	AUPR
1	0.7596	0.7470	0.7519	0.749	0.8863	0.8321
2	0.7592	0.7469	0.7479	0.7474	0.8762	0.8152
3	0.7853	0.7701	0.7857	0.7746	0.8936	0.8384
4	0.7791	0.7686	0.7839	0.7717	0.8942	0.8316
5	0.7822	0.7664	0.7798	0.7707	0.8946	0.8304
Mean	0.7731	0.7598	0.7698	0.7627	0.8890	0.8295
std	0.0113	0.0106	0.0164	0.0119	0.0071	0.0077

The results presented in Table 1 and Figure 2 demonstrate that BCheM achieved highly efficient predictive performance across all evaluation criteria in the 5-fold CV based on the benchmark dataset. Specifically, the average values for Acc, Prec., Rec., and F1-score reached 0.7731, 0.7598, 0.7698, and 0.7627, respectively, all exceeding 0.75. The average AUC and AUPR values were 0.8890 and 0.8295, respectively. Furthermore, the standard deviations for all these metrics in the five-fold cross-validation were 0.0113, 0.0106, 0.0164, 0.0119, 0.0071, and 0.0077, respectively. This indicates that BCheM can accurately predict drug resistance for multiple types of diseases and exhibits high stability in predictions across different subsets.

## 7 Validation of the molecular attribute understanding module

**FIGURE 3:**
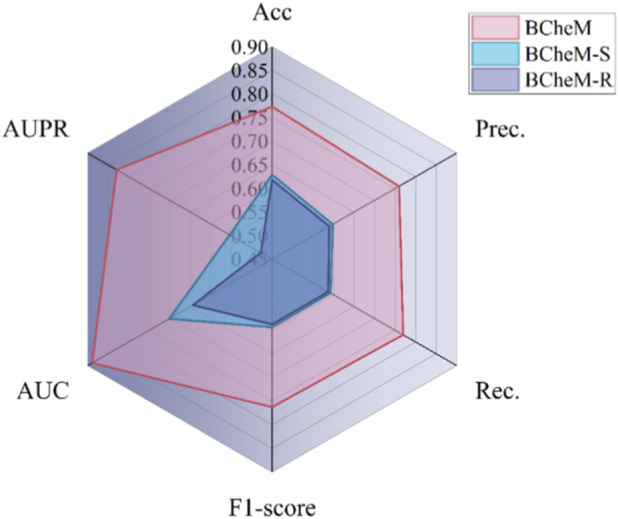
Performance comparison of BCheM variants across multiple evaluation metrics.

BCheM constructs a molecular attribute understanding module that leverages NLP techniques to learn hidden features in molecular attributes from both disease semantic descriptions and drug SMILES. This provides insights into the initial attributes of nodes for downstream disease-drug resistance graph modeling. To validate the effectiveness of the molecular attribute understanding module, we set up comparative experiments. Specifically, we devised molecular attribute extraction strategies from three perspectives: molecular attribute understanding, molecular attribute topology (SVD decomposition), and random initialization. Based on these perspectives, we constructed three models: BCheM, BCheM-S, and BCheM-R. We then used these three models to perform prediction tasks on benchmark datasets, with experimental results recorded in Table 2, and their corresponding visualizations are presented in Figure 3.

**TABLE 2:** Prediction results of BCheM based on different molecular attribute extraction perspectives.

BCheM	Acc	Prec.	Rec.	F1-score	AUC	AUPR
1	0.7596	0.7470	0.7519	0.749	0.8863	0.8321
2	0.7592	0.7469	0.7479	0.7474	0.8762	0.8152
3	0.7853	0.7701	0.7857	0.7746	0.8936	0.8384
4	0.7791	0.7686	0.7839	0.7717	0.8942	0.8316
5	0.7822	0.7664	0.7798	0.7707	0.8946	0.8304
Mean	0.7731	0.7598	0.7698	0.7627	0.8890	0.8295
std	0.0113	0.0106	0.0164	0.0119	0.0071	0.0077

BCheM-S	Acc	Prec.	Rec.	F1-score	AUC	AUPR
1	0.6263	0.5978	0.5896	0.5906	0.7083	0.5709
2	0.6242	0.5993	0.5939	0.5951	0.6882	0.5222
3	0.6411	0.6083	0.6061	0.6070	0.6877	0.5252
4	0.6227	0.5871	0.5798	0.5809	0.7016	0.5657
5	0.6319	0.5968	0.5933	0.5945	0.7202	0.5822
Mean	0.6292	0.5979	0.5925	0.5936	0.7012	0.5532
std	0.0067	0.0068	0.0085	0.0084	0.0124	0.0247

BCheM-R	Acc	Prec.	Rec.	F1-score	AUC	AUPR
1	0.6309	0.607	0.6037	0.6048	0.666	0.5006
2	0.6089	0.5838	0.5806	0.5814	0.6351	0.5008
3	0.6074	0.5788	0.5807	0.5794	0.6393	0.4601
4	0.6227	0.5935	0.5915	0.5923	0.6456	0.4855
5	0.6227	0.5837	0.5787	0.5799	0.6309	0.4414
Mean	0.6185	0.5894	0.5870	0.5876	0.6434	0.4777
std	0.0090	0.0100	0.0095	0.0098	0.0123	0.0234

As shown in Table 2 and Figure 3, BCheM, equipped with the molecular attribute understanding module, achieves the best performance across all evaluation metrics, significantly outperforming both the SVD-based variant (BCheM-S) and the randomly initialized variant (BCheM-R). Specifically, BCheM attains AUC and AUPR values of 0.8890 and 0.8295, respectively, which are substantially higher than those of BCheM-S (AUC = 0.7012, AUPR = 0.5532) and BCheM-R (AUC = 0.6434, AUPR = 0.4777). These results demonstrate that the proposed molecular attribute understanding module effectively enhances the model’s discriminative ability and generalization performance.

From the perspective of feature construction strategies, BCheM leverages pre-trained models to encode disease semantic descriptions and drug SMILES representations, enabling the extraction of both semantic and structural information at the molecular level. This domain knowledge-driven representation learning approach facilitates the capture of complex and latent associations between diseases and drugs, thereby providing high-quality input features for subsequent graph modeling. As a result, BCheM exhibits more stable and superior performance across all evaluation metrics.

In contrast, BCheM-S employs SVD decomposition to extract features from the network topology. Although this approach can achieve dimensionality reduction and noise filtering to some extent, it is inherently an unsupervised linear representation method, which limits its ability to model nonlinear relationships and domain-specific semantic information. Consequently, its performance in terms of Precision, Recall, and F1-score remains relatively inferior. Meanwhile, BCheM-R utilizes randomly initialized features that lack any structural or semantic information, making it difficult for the model to learn meaningful discriminative patterns, resulting in the poorest overall performance.

Overall, these findings highlight the critical role of the molecular attribute understanding module in the proposed framework. By effectively integrating disease semantic information and drug structural information, BCheM is able to generate more informative and discriminative feature representations, thereby significantly improving prediction accuracy and robustness in complex multimodal data scenarios.

## 8 Effectiveness of the attention mechanism

BCheM models the drug-disease resistance association with initial attribute features *via* graph-level modeling using Chebyshev convolution with multi-head attention mechanisms. By incorporating multi-head attention, BCheM allows for assigning different weights to different node pairs, enabling the model to capture relationships between nodes in various contexts, thus enhancing prediction performance. In this section, we validate the effectiveness of the attention mechanism through ablation experiments. Specifically, we constructed models BCheM-N, BCheM-1, and BCheM by employing Chebyshev convolution without attention, with single-head attention, and with two-head attention, respectively. We performed the 5-fold CV on benchmark datasets for these models to evaluate their performance with different attention mechanisms. The results of the ablation and comparison experiments are documented in Table 3.

**TABLE 3:** Prediction results of BCheM with different attention mechanisms.

BCheM-N	Acc	Prec.	Rec.	F1-score	AUC	AUPR
1	0.7351	0.7204	0.7187	0.7195	0.8585	0.7908
2	0.7515	0.7396	0.731	0.7343	0.8756	0.8020
3	0.7607	0.7407	0.7433	0.7419	0.8793	0.8059
4	0.7469	0.7294	0.729	0.7292	0.8773	0.8087
5	0.7423	0.7208	0.7156	0.7179	0.8788	0.8013
Mean	0.7473	0.7302	0.7275	0.7286	0.8739	0.8017
std	0.0086	0.0088	0.0098	0.0090	0.0078	0.0061

BCheM-1	Acc	Prec.	Rec.	F1-score	AUC	AUPR
1	0.7626	0.7507	0.7573	0.7531	0.8859	0.8318
2	0.7761	0.7646	0.7653	0.7649	0.8864	0.8256
3	0.7546	0.7365	0.7469	0.7401	0.8849	0.8244
4	0.7669	0.7545	0.7674	0.7578	0.8849	0.8203
5	0.7638	0.7465	0.7572	0.7502	0.8795	0.7750
Mean	0.7648	0.7506	0.7588	0.7532	0.8843	0.8154
std	0.0070	0.0092	0.0072	0.0082	0.0025	0.0205

BCheM	Acc	Prec.	Rec.	F1-score	AUC	AUPR
1	0.7596	0.7470	0.7519	0.7490	0.8863	0.8321
2	0.7592	0.7469	0.7479	0.7474	0.8762	0.8152
3	0.7853	0.7701	0.7857	0.7746	0.8936	0.8384
4	0.7791	0.7686	0.7839	0.7717	0.8942	0.8316
5	0.7822	0.7664	0.7798	0.7707	0.8946	0.8304
Mean	0.7731	0.7598	0.7698	0.7627	0.8890	0.8295
std	0.0113	0.0106	0.0164	0.0119	0.0071	0.0077

In the results presented in Table 3, the BCheM model attained an Acc of 0.7731, significantly higher than the 0.7648 of BCheM-1 and 0.7473 of BCheM-N, indicating that the 2-head attention mechanism enhances overall prediction accuracy. In terms of Prec., the BCheM model achieved 0.7598, surpassing BCheM-1’s 0.7506 and BCheM-N’s 0.7302, thus reducing false positive rates and increasing predictive reliability, which is crucial in drug-response scenarios, thereby mitigating the high costs associated with false alarms. Regarding Rec., the BCheM model reached 0.7698, compared to 0.7588 for BCheM-1 and 0.7275 for BCheM-N, suggesting heightened efficacy in identifying drug resistance and minimizing false negatives. The BCheM model also exhibited superior performance in the F1-score, scoring 0.7627 against BCheM-1’s 0.7532 and BCheM-N’s 0.7286. In the AUC, the BCheM model scored 0.8890, higher than BCheM-1’s 0.8843 and BCheM-N’s 0.8739, demonstrating stronger discriminatory abilities and less sensitivity to threshold selection. In the AUPR, the BCheM model’s 0.8295 was notably higher than BCheM-1’s 0.8154 and BCheM-N’s 0.8017, indicating its ability to maintain high precision across different recall levels when handling imbalanced datasets.

In summary, the BCheM model incorporating a 2-head attention mechanism significantly enhances the capture and understanding of complex relationships within disease-drug response graphs. The multi-head attention mechanism, by attending to different information channels, improves the diversity and accuracy of feature representations, resulting in superior performance across numerous key metrics. This not only boosts predictive capabilities but also enhances robustness and reliability in practical applications, confirming the crucial role of multi-head attention mechanisms in graph neural networks.

## 9 Optimal molecular feature extraction dimension

**FIGURE 4:**
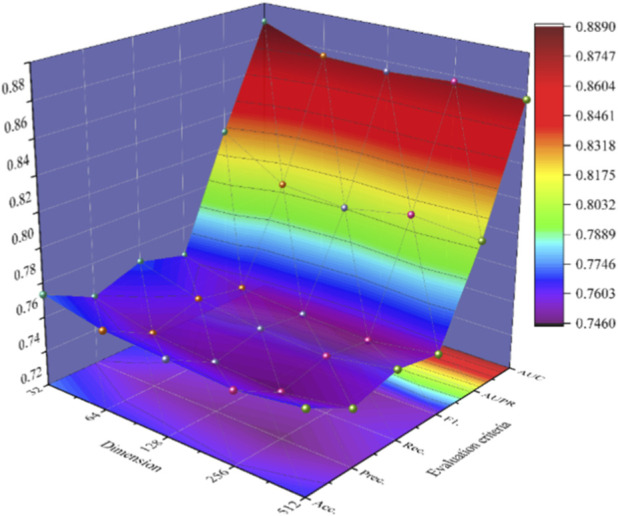
Three-dimensional analysis of the influence of feature dimensionality on BCheM performance.

BCheM integrates a molecular attribute understanding module and a Chebyshev graph convolutional model with a multi-head attention mechanism to extract molecular feature descriptors from molecular properties and disease-drug response relationship graphs. These descriptors are used for classifier training and prediction tasks in downstream classification. Proper selection of the feature dimension allows the model to focus on the most relevant information, thereby improving the model’s generalization ability and computational efficiency. Thus, choosing the right feature dimension is critical for achieving optimal performance in machine learning tasks. In this section, we conduct a comparative study to select the optimal feature extraction dimension for the model. Specifically, we extract 32, 64, 128, 256, and 512-dimensional molecular representations based on a benchmark dataset, and record the predictive results for each dimension using 5-fold CV. The prediction results are summarized in Figure 4.

The results presented in Figure 4 indicate that, although the model shows slight improvements in certain metrics with increased dimensionality, the 32-dimensional feature extraction BCheM model consistently outperforms across all evaluated metrics. Notably, the 32-dimensional feature extraction BCheM model demonstrates significant advantages in key performance metrics such as accuracy, precision, F1-score, and AUC. This performance not only enhances the predictive capability of the model but also ensures computational efficiency and reliability in practical applications. These findings suggest that, within the specific context of this study, 32-dimensional feature extraction represents a valuable and effective choice.

## 10 Comparison with advanced graph representation methods

To validate the rationality and superiority of the BCheM model, we compare BCheM with state-of-the-art (SOTA) methods from existing research to verify its performance advantages. As far as we know, only a limited number of studies have focused on drug response problems under multi-type disease backgrounds, providing few reference methods. Therefore, we selected SOTA models for similar tasks for comparison. In addition, to eliminate differences among tasks in different studies and ensure a fair comparison, we combined the core methods used in SOTA with BCheM to construct drug response prediction models suitable for multi-type disease backgrounds.

Specifically, in the miRNA-drug resistance prediction domain, Huang et al. proposed the GCMDR method, which utilizes graph convolution techniques (Kipf and Welling, 2016) to construct graph-level modeling of miRNA-drug resistance for downstream prediction tasks (Huang et al., 2020). For human microbe-drug association prediction, Ma et al. introduced the GACNNMDA model, which aggregates feature matrices and heterogeneous microbe-drug networks through a graph attention network (Velickovic et al., 2017) to obtain low-dimensional molecular feature representations (Ma et al., 2023). In the prediction of gene-bridged metabolite-disease relationships, Lu et al. proposed the MGDHGS framework, which uses GraphSAGE (Hamilton et al., 2017) to learn node neighborhood representations within the metabolite-gene-disease heterogeneous network to support downstream prediction tasks (Lu and Li, 2024). For molecular toxicity prediction, Liu et al. proposed MTBG, which extracts features from molecular graphs using GraphSAGE to predict unknown molecular toxicity (Liu et al., 2023). In circRNA-miRNA prediction, Yu et al. presented SGCNCMI, which combines graph convolutional neural networks with attention mechanisms to propagate multi-modal features of molecules and predict unknown circRNA-miRNA associations (Yu et al., 2022). Guo et al. proposed WSCD, which utilizes SDNE (Wang et al., 2016) to extract behavioral features within the CMI network to predict unknown CMI (Guo et al., 2022). These methods employ advanced algorithms to build models and achieve efficient prediction results. We combined the core algorithms of these methods with BCheM to construct new models for performance comparison. Specifically, we built new BCheM models by integrating GCN, attention-based GCN, GraphSAGE, GAT, and SDNE algorithms, resulting in BCheGCN, BCheSGCN, BCheSAGE, BCheGAT, and BCheSDNE, respectively. Additionally, to comprehensively compare advanced methods, we included BCheTF, BCheCHe, and BChe1H, which incorporate transformer convolution and varying numbers of attention heads (0, 1 attention heads). These methods were evaluated based on a benchmark dataset, and the results are recorded in Table 4.

**TABLE 4:** Prediction results of BCheM and SOTA methods.

Method	Acc.	Prec.	Rec.	F1.	AUC	AUPR
BCheGCN	0.7458 ± 0.0089	0.7286 ± 0.0096	0.7264 ± 0.0109	0.7271 ± 0.0100	0.8657 ± 0.0059	0.7971 ± 0.0133
BCheSGCN	0.7498 ± 0.0131	0.7329 ± 0.0131	0.7318 ± 0.0159	0.7320 ± 0.0143	0.8654 ± 0.0071	0.7490 ± 0.0090
BCheSAGE	0.7495 ± 0.0108	0.7327 ± 0.0104	0.7312 ± 0.0131	0.7316 ± 0.0117	0.8683 ± 0.0064	0.8016 ± 0.0084
BCheGAT	0.7403 ± 0.0068	0.7227 ± 0.0041	0.7225 ± 0.0066	0.7225 ± 0.0051	0.8687 ± 0.0071	0.8018 ± 0.0044
BCheSDNE	0.7455 ± 0.0126	0.7283 ± 0.0120	0.7282 ± 0.0144	0.7281 ± 0.0131	0.8597 ± 0.0089	0.7872 ± 0.0142
BCheTF	0.7504 ± 0.0083	0.7337 ± 0.0069	0.7344 ± 0.0086	0.7338 ± 0.0075	0.8618 ± 0.0058	0.7858 ± 0.0101
BCheCHe	0.7473 ± 0.0086	0.7302 ± 0.0088	0.7275 ± 0.0098	0.7286 ± 0.0090	0.8739 ± 0.0078	0.8017 ± 0.0061
BChe1H	0.7648 ± 0.0070	0.7506 ± 0.0092	0.7588 ± 0.0072	0.7532 ± 0.0082	0.8843 ± 0.0025	0.8154 ± 0.0205
BCheM	0.7731 ± 0.0113	0.7598 ± 0.0106	0.7698 ± 0.0164	0.7627 ± 0.0119	0.8890 ± 0.0071	0.8295 ± 0.0077

As shown in Table 4, BCheM achieves the best performance across all evaluation metrics, significantly outperforming all competing models, which demonstrates the effectiveness and superiority of the proposed method in multi-disease drug resistance prediction tasks. Specifically, BCheM attains the highest values in key metrics, including Acc, F1-score, AUC, and AUPR. Notably, it achieves an AUC of 0.8890 and an AUPR of 0.8295, indicating strong discriminative ability and robustness in handling class imbalance.

From the comparison of different models, it can be observed that traditional graph convolution and network embedding-based methods (e.g., BCheGCN, BCheSAGE, and BCheSDNE) generally exhibit relatively lower performance. This is mainly because such methods tend to focus on local structural information or single-type feature modeling, making it difficult to effectively capture the complex multimodal relationships between diseases and drugs. In particular, the inferior performance of BCheSDNE suggests that relying solely on network structure embeddings is insufficient to model complex biological data that involve both semantic information and molecular structural features.

In addition, although the Transformer-based convolution model (BCheTF) has advantages in modeling long-range dependencies, its performance does not surpass that of traditional graph convolution methods. This may be attributed to the fact that drug response prediction tasks rely more on local structural information and multi-order neighborhood features rather than long-range dependencies, thereby limiting the effectiveness of Transformer-based approaches in this context. Notably, models based on Chebyshev convolution (e.g., BCheCHe, BChe1H, and BCheM) consistently outperform other graph convolution methods, especially in terms of AUC and AUPR. This indicates that Chebyshev convolution, through polynomial approximation, is capable of effectively capturing higher-order neighborhood information. Compared with traditional GCN models that rely primarily on first-order neighborhoods, it is more effective in extracting complex graph structural features, thereby enhancing the model’s representational capacity. Furthermore, its parameter-sharing mechanism not only improves computational efficiency but also helps mitigate overfitting, leading to better generalization performance.

Building upon this, BCheM further incorporates a multi-head attention mechanism to adaptively assign weights to different node relationships. This enables the model to capture latent associations between diseases and drugs from multiple subspaces, facilitating deep integration of attribute features and structural information. As a result, compared with models using single-head attention or no attention mechanism (e.g., BChe1H and BCheCHe), BCheM achieves further improvements across all evaluation metrics.

In summary, by integrating molecular attribute understanding with multi-head attention-based graph modeling, BCheM demonstrates significant advantages in modeling multimodal information and complex relational structures. This enables it to achieve the best performance in multi-disease drug resistance prediction tasks, highlighting its strong competitiveness and practical applicability.

## 11 Case study

To verify the practicality of BCheM in predicting disease-drug resistance responses, we conducted case studies on two important drugs in cancer treatment: Fluorouracil and Doxorubicin. Fluorouracil is an antimetabolite that belongs to the class of pyrimidine analogs. It inhibits the growth of cancer cells by interfering with the synthesis of DNA and RNA. Fluorouracil is primarily used to treat various types of cancer, including colorectal cancer, breast cancer, gastric cancer, pancreatic cancer, and head and neck cancers (Van Cutsem et al., 2015). Doxorubicin is an anthracycline antibiotic with a broad spectrum of antitumor activity. It exerts its effects by intercalating into the DNA double helix, disrupting DNA replication and transcription, thereby inhibiting the division and proliferation of cancer cells. Doxorubicin is widely used in the treatment of various cancers, including breast cancer, ovarian cancer, lung cancer, lymphomas, and leukemias (Carvalho et al., 2009).

Specifically, we trained on known data using BCheM to predict the likelihood of drug resistance for Fluorouracil and Doxorubicin across diseases present in the dataset. A selection of diseases with a high potential for resistance was made, and the results of the case study are documented in Table 5.

**TABLE 5:** Results of case study on drug resistance of Fluorouracil and Doxorubicin.

Num	Drug	Disease	Response	Evidence (PMID)
1	Fluorouracil	Gastric cancer	Resistance	29,162,158
2	Fluorouracil	Colorectal cancer	Resistance	21078976
3	Fluorouracil	Liver cancer	Resistance	20978511
4	Fluorouracil	Esophageal cancer	Resistance	27501171
5	Fluorouracil	Cervical cancer	Resistance	Unconfirmed
6	Fluorouracil	Breast cancer	Resistance	22523546
7	Fluorouracil	Oral squamous cell carcinoma	Resistance	29364705
8	Fluorouracil	Pancreatic cancer	Resistance	25117811
9	Fluorouracil	Kidney cancer	Resistance	31754403
10	Fluorouracil	Lung cancer	Resistance	22193543
11	Doxorubicin	Bacterial infection	Resistance	21468550
12	Doxorubicin	Osteosarcoma	Resistance	28323030
13	Doxorubicin	Pneumonia	Resistance	16189122
14	Doxorubicin	Melanoma	Resistance	Unconfirmed
15	Doxorubicin	Leiomyosarcoma	Resistance	33685462
16	Doxorubicin	Diffuse large B-cell lymphoma	Resistance	29966970
17	Doxorubicin	Lactic acidosis	Resistance	32573588
18	Doxorubicin	Mycosis fungoides	Resistance	32795528
19	Doxorubicin	Acute myeloid leukemia	Resistance	Unconfirmed
20	Doxorubicin	Peripheral nerve sheath tumor	Resistance	22664653

The case study results indicate that among the ten diseases with the high probability of resistance predicted by BCheM for Fluorouracil, nine were confirmed by known clinical reports. For Doxorubicin, eight out of the ten predicted diseases were confirmed by clinical reports. Notably, the unconfirmed results do not imply inaccuracy but rather represent potential diseases with high resistance. The prediction results based on drug resistance for both anticancer drugs demonstrate that BCheM can effectively predict the potential drug resistance of diseases, making it a promising candidate for a computational tool to predict disease resistance.

## 12 Discussion

The response of diseases to drugs plays a critical role in personalized medicine. Although existing studies have made progress in specific disease contexts, most of them lack a unified modeling framework for capturing complex relationships across multiple diseases. To address this issue, we construct a multi-disease drug resistance dataset and propose the BCheM framework, which integrates molecular attribute information with graph structural relationships to effectively predict drug resistance. Experimental results and case studies demonstrate that the proposed method outperforms existing approaches across multiple evaluation metrics, confirming its effectiveness and practical potential.

Despite these promising results, several limitations remain. First, the semantic descriptions of diseases rely on large language models, which may introduce potential bias. Second, the current model is based on static relationship modeling and does not account for the dynamic nature of drug responses. In addition, the modeling of fine-grained biological mechanisms remains insufficient, which may limit the interpretability and generalization ability of the model.

From an applicability perspective, BCheM is more suitable for drug resistance prediction tasks in multi-disease scenarios with relatively rich data support, while its performance in data-sparse or cross-domain settings requires further investigation. Future work may focus on incorporating multi-omics data, developing dynamic graph modeling approaches, and enhancing model interpretability to further improve predictive performance and clinical applicability.

## Data Availability

Publicly available datasets were analyzed in this study. This data can be found here: https://github.com/Yuu088/BCheM.
